# Could enteral nutrition improve the outcome of patients with haematological malignancies undergoing allogeneic haematopoietic stem cell transplantation? A study protocol for a randomized controlled trial (the NEPHA study)

**DOI:** 10.1186/s13063-015-0663-8

**Published:** 2015-04-07

**Authors:** Richard Lemal, Aurélie Cabrespine, Bruno Pereira, Cécile Combal, Aurélie Ravinet, Eric Hermet, Jacques-Olivier Bay, Corinne Bouteloup

**Affiliations:** CHU Clermont-Ferrand, Service d’Hématologie Clinique Adulte et de Thérapie Cellulaire, F-63003 Clermont-Ferrand, France; Clermont Université, Université d’Auvergne, EA7283, CIC501, BP 10448, F-63000 Clermont-Ferrand, France; CHU Clermont-Ferrand, Unité biostatistique Direction de la Recherche Clinique, F-63003 Clermont-Ferrand, France; CHU Clermont-Ferrand, Service Diététique, F-63003 Clermont-Ferrand, France; CHU Clermont-Ferrand, Service de Médecine Digestive et Hépatobiliaire, F-63003 Clermont-Ferrand, France; Clermont Université, Université d’Auvergne, Unité de Nutrition Humaine, BP 10448, F-63000 Clermont-Ferrand, France; INRA, UMR 1019, UNH, CRNH Auvergne, F-63000 Clermont-Ferrand, France

## Abstract

**Background:**

Myeloablative allogeneic haematopoietic stem cell transplantation (allo-HSCT) is a major procedure usually accompanied by multifactorial malnutrition, prompting the recommendation of systematic artificial nutritional support. Parenteral nutrition (PN) is usually administered during allo-HSCT, essentially for practical reasons. Recently published data suggest that enteral nutrition (EN), given as systematic artificial nutrition support, could decrease grade III–IV graft-versus-host disease (GVHD) and infectious events, which are associated with early toxicity after allo-HSCT and then have an impact on early transplant-related mortality (D100 mortality).

**Methods/Design:**

We report on the NEPHA trial: an open-label, prospective, randomised, multi-centre study on two parallel groups, which has been designed to evaluate the effect of EN compared to PN on early toxicity after an allo-HSCT procedure. Two hundred forty patients treated with allo-HSCT for a haematological malignancy will be randomly assigned to two groups to receive either EN or PN. The primary endpoint will assess the effect of EN on D100 mortality. Secondary endpoints will compare EN and PN with regards to the main haematological, infectious and nutritional outcomes.

**Discussion:**

The impacts of nutritional support should exceed the limits of nutritional status improvement: EN may directly reduce immunological and infectious events, as well as decrease early transplant-related morbidity and mortality. EN and PN need to be prospectively compared in order to assess their impacts and to provide treatment guidelines. (Clinical trials gov number: NCT01955772; registration: July 19th, 2013).

## Background

Allogeneic haematopoietic stem cell transplantation (allo-HSCT) is a major procedure, and is usually conducted to consolidate remission of haematological malignancies. Allo-HSCT includes administration of a chemotherapy-based conditioning regimen (myelo-ablative or non-myelo-ablative), followed by infusion of alloreactive haematopoietic stem cells, with the aim of inducing an active immunological anti-tumoral effect. In cases where there is a myelo-ablative-conditioning regimen, drug-induced toxicities, immunosuppression-induced infections and acute graft-versus-host disease (GVHD) are responsible for 15–25% of early mortalities (D100 mortality) [1].

Cytotoxic drug toxicities are responsible for the quasi-systematic and steep decrease in spontaneous oral intake, that was shown to be associated with digestive GVHD [2,3]. In the absence of artificial nutritional support, malnutrition can occur quickly; hypercatabolism, multiple and invasive treatments, and their complications (infection, inflammation) [4], increase malnutrition. Several studies show that malnutrition is an independent negative prognostic factor for the survival of children and adults affected by malignant haematological disease and treated by allo-HSCT [5-8]. Furthermore, malnutrition decreases quality of life [9] and increases length-of-stay in hospital [10].

The American Society of Parenteral and Enteral Nutrition (ASPEN) and the French-speaking society of clinical nutrition and metabolism (SFNEP) recommend nutritional support during haematopoietic transplantation for patients who are malnourished or have decreased intake or decreased intestinal absorption over a prolonged period (grade B) [11,12]. For practical and historical reasons, parenteral nutrition (PN) is often the first option chosen for patients undergoing an allo-HSCT [13]. Indeed, allo-HSCT patients all have a central-venous access that facilitates PN administration. PN has been shown to be safe and effective and to improve nutritional state. It preserves body mass during HSCT [14-16], and increases 2-year overall survival and relapse-free survival [17]. Nevertheless, in numerous clinical settings with hypermetabolism, including cancer and for patients in Intensive Care Units (ICUs), PN is associated with a larger number of complications, mostly infectious [18,19].

In allo-HSCT, PN, compared to simple hydration, increased infectious complications, which led to an increase in early morbidity and mortality, and subsequent increased costs and hospital length-of-stay [14,20]. Enteral nutrition (EN) has been shown to be feasible in small retrospective cohorts of paediatric and adult allo-HSCT patients [21,22]. However, the nasogastric tube remains poorly perceived by caring teams and patients, and is thought to be traumatic and uncomfortable. However, Seguy et al. [23] reported on a non-randomised study that included a small cohort of 45 allo-HSCT patients receiving a myelo-ablative conditioning regimen: they showed that EN decreased the incidence of grade III/IV GVHD and infection-related mortality at D100. The same team recently published their results on a larger cohort of 121 monocentric consecutive allo-HSCT patients that received a myelo-ablative conditioning regimen, and confirmed the same benefits for EN vs PN, with a protective effect of EN on early overall survival, on infectious mortality and on the incidence of grade III/IV acute GVHD [24].

We have recently reported our retrospective experience, showing that EN was associated with a lower risk of early infectious complications, but had no impact on the incidence of GVHD or on D100 survival in 56 consecutive patients who received a myelo-ablative or non-myelo-ablative conditioning regimen [25].

Until now, no prospective randomised study has confirmed the benefits of EN in allo-HSCT patients. This lack of solid proof negatively impacts on the development of EN use.

## Methods and design

We plan to conduct a randomised, controlled, prospective trial to determine the best nutritional support modalities for allo-HSCT patients.

### Objectives

The main objective is to evaluate the effect of EN compared to PN on early mortality (at day 100 [D100]) in patients treated with myeloablative allo-HSCT.

The main secondary objectives are to evaluate the effects of EN compared to PN on:

- overall survival and progression-free survival at 1 year;
- haematologic evolution: i.e. incidence and severity of GVHD, secondary toxicities (namely, infections and mucositis), and haematopoietic reconstitution and engraftment;
- nutritional parameters: i.e. nutritional and functional status, duration of nutritional support before recovery to oral feeding, nutritional support tolerance (in particular at an infectious, digestive and hepatobiliary level);
- assessment of quality of life.

### Study design and location

**Figure 1:**
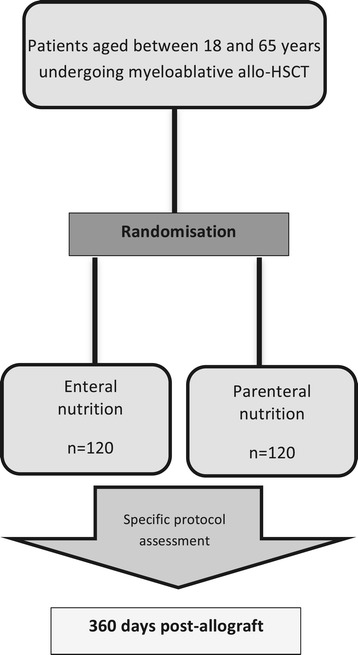
**Flow chart describing the study’s design.**

NEPHA is an open-label, controlled, prospective, randomised, multi-centre clinical study. It will enrol 240 patients, who will be distributed randomly between the following two groups according to the type of nutritional support, EN or PN (Figure 1):

- the EN group will represent patients receiving EN via a nasogastric tube;
- the PN group will represent patients receiving PN via a central venous line.

### Study population and ethical aspects

The patients will be recruited in each participating centre by the investigating haematologist during the pre-transplantation consultation. Inclusion and exclusion criteria are described in Table 1. All patients will have provided their informed consent, and the study will be conducted according to the principles of the Declaration of Helsinki. Local ethical committee agreement was obtained in December 2012 (Comité de Protection des Personnes (CPP) Sud-Est VI; reference 2011-A01288-33); French National Health Authorities agreement was obtained in July 2013 (Agence Nationale de Sécurité du Médicament et des produits de santé (ANSM); reference 130334B-42).

**Table 1:** **Inclusion and exclusion criteria**

**Inclusion criteria**	- Aged between 18 and 65 years
	- Men and women
	- 2Patients undergoing myeloablative allo-HSCT for a haematological malignancy
	- HLA-compatibility: geno-identical or pheno-identical 10/10
	- Patients affiliated with a social-security organisation
	- Patients had signed the informed consent
**Exclusion criteria**	- Status of tumour progression at the moment of the allo-HSCT
	- HLA compatibility ≤ 9/10
	- Artificial nutrition used at the moment of inclusion
	- Inability to understand the protocol (linguistic barrier, cognitive difficulties)
	- Contraindication or associated pathology that does not allow us to carry out EN or PN according to the protocol
	- Medical history of progressive psychiatric illness
	- Medical history of another progressive cancer or occurrence in the 5 previous years
	- Presence of a simultaneous serious and uncontrolled disease, such as severe cardiac, renal, hepatic or respiratory failure, or severe sepsis
	- Previous allo-HSCT
	- Antibiotic use for digestive decontamination
	- Participation in another clinical trial studying an allograft procedure, and applying modalities that are not available in routine practice (including innovative immunosuppression and graft or conditioning regimens not considered as myeloablative).

### Randomisation

Randomisation will be centralised in Clermont-Ferrand University Hospital. It will be stratified by centre in order to take the 'centre effect' into account (especially for conditioning modalities, prevention and treatment of infectious complications). We supposed that this center stratification will permit to correctly balance the two groups regarding local specific therapeutic habits. Moreover, we assume that conditioning regimens and drugs administered as prophylactic regimens towards GVHD will not be imbalanced between the two groups : indeed, inclusion and exclusion criteria enable to focus the trial on patients with high HLA-compatibility, receiving a myeloablative conditioning regimen without innovative intervention.

Randomisation will be conducted to balance group sizes according to a computer-generated allocation sequence by blocks with Clinsight® software package.

### Nutritional intervention

EN or PN will be started systematically at D1 (maximum at D2) after transplantation (D0 being the day of transplantation), without taking oral intake into account. The two groups will be isocaloric, isonitrogenous with energy and nitrogen intake targets fixed respectively at 30–35 kcal and 1.2–1.5 g protein per kg per day. During the first 14 days, all patients will receive an intravenous glutamine supplementation of 0.3 g per kg per day (Dipeptiven® [dipeptide alanyl-glutamine], Fresenius Kabi).

For patients in the EN group, a polyurethane or silicone nasogastric tube, 8- to 10-French, will be inserted and its correct positioning will be controlled by radiography before starting EN. The EN will be then introduced prudently with flow and volume progressively increased until the target intake is reached in 7 days, following a pre-established and validated protocol. This procedure will be carried out with a flow regulator in nocturnal cyclical mode over 10–16 hours, unless there is a justified contraindication (in particular, a high risk of inhalation).

Initially, the infused nutritive mixtures will be polymeric, normo- or hypercaloric, normo- or hyperprotidic, and without immuno-modulating substrates. Fibre-containing mixtures and semi-elemental mixtures will be allowed according to EN digestive tolerance and the medical context (as is usually proposed). All nutritive mixtures used will be supplied by NUTRICIA Medical Nutrition Laboratory.

If there is any digestive intolerance (diarrhoea, vomiting, cases of acute GVHD), EN will be maintained if possible, and the flow and volume will be adapted as necessary. If the protein energetic-target intake is not obtained, and has a deficit of >500 kcal per day for more than 48h, supplementary PN will be added to bridge the gap. Except for refusal by the patient or insertion failure, the tube will be reinserted up to a minimum of three times before ceasing EN. Any reason for stopping EN during the follow-up (and replacement with PN) will be recorded by the investigator. These events will help us to evaluate tolerance to EN.

For patients in the PN group, PN will be administrated by a central venous catheter, which is usually inserted into allo-HSCT patients to allow administration of chemotherapy and different parenteral treatments. A standard three-in-one mixture will be used, according to the products usually used in each centre. In order to avoid heterogeneity in PN modalities, the use of a three-in-one mixture or a lipid emulsion that contains omega-3 fatty acids will be disallowed, unless the centre has no other available products. The usual recommended vitamin and trace-element supplements will be given.

Unless there is a justified contraindication (risk of overload, glycaemia that is difficult to control…), PN will be conducted in a nocturnal cyclical mode over a minimum of 12 hours, and will be based on overall volume and glucose-infusion rate (<4 mg per kg per min). The cyclical administration of PN will limit the risks of hepatobiliary complications related to PN *per se*.

Spontaneous oral feeding will be maintained as necessary and according to the patient’s appetite. Evaluation of daily oral intake will be carried out by the dietician. Hypercaloric, hyperprotidic oral nutritional supplements, without immuno-modulating substrates, will be authorised. These will be recorded by the dietician and included in the total oral-intake calculation.

EN or PN will be decreased when the patient has resumed oral feeding that allows him/her to obtain 50% of his/her daily needs (15–18 kcal per kg per day), and will be stopped when 75% of his/her needs are satisfied (22.5–26.0 kcal per kg per day).

### Haematological treatment

The recruiting services participating in the study are authorised to perform allo-HSCT. The terms of conditioning, the prevention of GVHD (including the use of anti-thymoglobulin, mycophenolate mofetil and methotrexate) and the management of toxicities will be done accordingly to the usual procedures at the different centres. All medications that are necessary after transplantation and/or for complications will be given as usual modalities at each centre, whatever the treatment group: EN or PN. Before admission to the allo-HSCT procedure, a biological profile, adapted to the patient’s pathology and according to the usual practices, will be performed. The transplant will be injected at D0 (day of the first injection). Participation in this study will not modify the usual clinical and biological follow-up protocols.

### Evaluation of outcomes

The primary endpoint evaluated will be mortality rate (proportion of deaths), observed at D100 in both groups. In the usual manner, early toxicities from allo-HSCT will be evaluated to see if mortality was related to the transplant at D100, which is considered as a dichotomous parameter without expected right-censored data [1]. D100 mortality is a strong marker of early allo-HSCT toxicity, classically used in allo-HSCT trials.

Secondary criteria will define the impact of the nutrition procedure on both transplantation morbidity/mortality and nutritional outcomes. Table 2 summarises the data-collection schedule.

**Table 2:** **Schedule of visits and assessment**

	**Time point**										
	**D-14 to D-7**	**D1**	**D7**	**D14**	**D21**	**D28**	**D60**	**D90**	**D180**	**D270**	**D360**
**Informed consent**	**x**										
**Allocation**	**x**										
**Energetic and caloric needs assessment**			**x**	**x**	**x**	**-------------------------------------➔** ^**1**^					
**Digestive symptoms and tolerance** (individual notebook)		**x**	**--------------------------------------------------------- ➔** ^**2**^								
**Functional status** (Performance status, sit-up test, dynamometry, Weight, body-mass index, brachial circumference)	**x**			**x**		**x**	**x**	**x**	**x**		**x**
**Hepatic tolerance** (Total bilirubin, free and conjugated bilirubin, AST, ALT, ALP, GGT)	**x**		**x**	**x**	**x**	**x**	**x**	**x**	**x**	**x**	**x**
**Transthyretin, C-reactive protein**	**x**		**x**	**x**	**x**	**-------------------------------------➔** ^**3**^					
**Albumin**	**x**					**x**	**x**	**x**	**x**		**x**
**Clinical assessment** (GVHD, mucositis, infectious complications, red blood cells and platelets transfusions, catheter complications)			**x**	**x**	**x**	**x**	**x**	**x**	**x**	**x**	**x**
**Chimerism**						**x**	**x**	**x**	**x**		**x**
**Quality of life (QLQ-C30)**	**x**							**x**	**x**		**x**

^1^Evaluated until hospital discharge; ^2^Auto-evaluated daily during nutritional intervention; ^3^Evaluated once per week until hospital discharge; ALP: alkaline phosphatase; ALT: alanine aminotransferase; AST: aspartate aminotransferase; GGT: gamma-glutamyltransferase.

Such iterative and multiparametric evaluations are usually done and reported in all allo-HSCT trials. The three retrospective studies published in this specific "enteral nutrition / allo-HSCT" field(23–25) used the same methodology and criteria in order to correctly assess allo-HSCT complex and dynamic issues.

### Transplantation morbidity and mortality

Most criteria will be evaluated according to routinely assessed parameters, without increasing the complexity of the usual allo-HSCT procedures.

Overall survival will be defined as the time period between the date of allogeneic transplantation (D0) and the date of death, regardless of its cause, and will be evaluated at 1 year.

Progression-free survival will be defined as the time period between the date of allogeneic transplantation (D0) and the date of disease progression or death (regardless of its cause), whichever comes first. This will also be evaluated at 1 year.

GVHD occurrence will be notified by specifying the location (liver, skin, gut), the Glucksberg severity score [26], the treatment applied and the efficacy of treatment. It will be notified every week up until D28 (minimum) or until hospital discharge, then monthly until D180, and then at D270 and D360.

Mucositis occurrence will be clinically evaluated every day up to D28, and will focus on its grade (according to the NCI-CTC criteria), the treatment applied and its duration.

Infectious complications will be evaluated every week up to D30 (minimum) or until hospital discharge, then every month until D180, and then at D270 and D360 according to the following.

- The existence of a documented bacteraemia (in the event of coagulase-negative *Staphylococcus bacteraemia*, two positive haemocultures will be necessary to retain a diagnosis of significant bacteraemia) and the number of days of curative antibiotherapy.
- The existence of a documented fungal infection and the type and number of days of preventive and curative antifungal treatment. The diagnosis of invasive pulmonary aspergillosis will be valued according to the EORTC/MSG 2008-revised criteria (possible, probable or proven) [27].
- The existence of a documented viral infection and the type and the number of days of curative antiviral treatment.
- The number of days with a fever (>38°); these episodes are frequent and not always linked to a documented infection. However, in these neutropenic and immunocompromised patients, fever is considered to be a strong surrogate marker for infection.

Infectious complications will also be evaluated indirectly by assessing two events, which usually are almost exclusively related to infections within the allo-HSCT setting:

- The need for an ICU transfer, with severity evaluated using the following criteria: 1) clinically relevant and routinely used severity indexes (SOFA index, IGS2 score, D28 ventilator-free delay); 2) invasive ventilation and/or non-invasive need for ventilation; 3) extra-renal purification or need for dialysis; 4) length of stay in the ICU.
- The need to remove a central catheter.

Haematopoietic reconstitution will be specified using daily full blood-count results. Haematopoietic reconstitution will be valued by:

- Turnaround time of polynuclear neutrophils > 0.5 × 10^9^/L (first day within a period of 3 consecutive days).
- Spontaneous platelet turnaround time: >20 × 10^9^/L (2 days with no platelet transfusion within the previous 3 days).
- Spontaneous platelet turnaround time: >50 × 10^9^/L (2 days with no platelet transfusion within the previous 3 days).
- The number of transfusions of red blood cells and platelets between D0 and D100.

Engraftment rates will be evaluated by measuring chimerism (determined by molecular biology, i.e. analysis of variable non-tandem repeat segments, or sexual chimerism by FISH in the event of sex mismatches) at D30, D60, D90 and D180. This evaluation is usual in allo-HSCT follow ups

### Nutritional outcomes

These criteria are usually not evaluated during the allo-HSCT procedure (except for weight and biological criteria), but will be specifically required for this prospective trial.

Nutritional status will be evaluated from clinical and biological criteria:

- Weight, body mass index and brachial circumference measured at D–7(*i.e*.7 days before treatment), once a week up to D28 or until hospital discharge, then once per month up to D180, and then at D270 and D360.
- Transthyretin and C-reactive protein (at D–7, then once a week during hospitalisation), albumin and C-reactive protein (at D–7, D7, D28, D60, D90, D180, D270 and D360).

Functional capacity (reflecting general condition and muscular efficiency, which can be easily linked to nutritional status) will be evaluated using the World Health Organisation Performance Status (0–4) and using the muscle strength measurement (on the upper limbs using a handgrip dynamometer, and on the lower limbs using the sit-up test) at D–7, D14, D28, D60, D90, D180 and D360.

The duration of the nutritional intervention, before resuming adequate oral feeding, will be defined by the number of days since the first day of EN or PN according to the randomisation group and the day of cessation of EN or PN (*i.e.* the patient had resumed oral feeding and was obtaining 75% of his/her protein-energetic needs). If patients in the EN group require PN supplementation, the duration of PN will be evaluated and compared with the PN group.

According to our previous unpublished experience, we do the assumption that morphometric measures and biological markers sould show a better nutritional status in EN group. In correlation with these results, functional status should be better in the EN group.

The randomized nutrition procedure is usually administered, on average, during 21 to 28 days: thus, we assume that improvement of nutritional status in EN group should be more relevant within the first 2 to 3 months after allo-HSCT, thereafter the difference between the two groups should be less important.

Nutritional support tolerance will be evaluated:

- Digestive symptoms will be evaluated daily with a focus on intensity of abdominal pain and intensity of nausea, using the Visual Analog Scale (0–100 mm), according to the number of vomiting events and number and consistency of stools. These criteria will be self-reported daily in an individual notebook by the patient, and will be overseen by nurses or dieticians. Moreover, weekly needs for symptomatic treatments will be assessed until the hospital discharge
- Hepatic tolerance will be evaluated by liver function tests twice a week until hospital discharge, then every 14 days until D90, and then at D180, D270 and D360 (total bilirubin, free and conjugated bilirubin, alkaline phosphatase, alanine aminotransferase, aspartate aminotransferase, gamma-glutamyl-transferase).
- Anomalies in glyco-regulation will be evaluated by a systematic daily venous glycemia test in both groups, and by capillary glycemia at the beginning and/or if there is modification to PN and, if necessary, in the EN group. Glycemia will be controlled by insulin therapy to avoid hyperglycaemic peaks, which are known to have a negative effect, and to increase complications and mortality, especially in ICUs.
- Mechanical and infectious/inflammatory complications caused by nasogastric tubes (patient refusal or local intolerance, insertion failures, falling out, obstructions, number of times nasogastric tube was inserted per patient, otitis, sinusitis…) and central venous catheters (occlusion, thrombosis, infection, number of successive catheters per patient) will be recorded.

Quality of life will be auto-evaluated by the patients using a validated questionnaire, (EORTC QLQ-C30 version 3), at D–7, D90, D180 and D360.

### Data management

In order to meet regulatory requirements (Guidance for Computerized systems Used in Clinical Trials, International Conference on Harmonisation, Good clinical Practice 2001/20/CE), e-Case Report Formdesign, data monitoring and database extractions will be performed with Clinsight® software package.

### Statistical considerations

#### Estimation of sample size

Sample-size estimation is based on the comparison of early mortality rates at day 100 for two-sided α-risk and β equal to 5% and 20%, respectively, and a difference between the two randomised groups of 12%. Indeed, D100 mortality rate in allo-HSCT patients receiving PN with intravenous glutamine supplementation was set at 17% (considering data from the literature [1,23-25]) and the D100 mortality rate in EN patients with intravenous glutamine supplementation was set at 5%. Under these assumptions and considering D100 mortality at least equal to or lower than Seguy et al.'s studies (in which no patient received glutamine), 240 subjects are needed to show the superior efficacy of EN over PN in terms of D100 mortality. Two interim analyses are planned at n = 80 and and n = 160 patients (inflation of type I-error using Lan et Demets, Obrien-Fleming, East©). An independent committee will advise on continuation of the study according to results from these two interim analyses.

### Statistical analysis

The analyses will be conducted as intention-to-treat with Stata software (version 13, Stata Corp, College Station, US). All statistical tests will be considered for a type-1 error α of 5% (except the interim analyses). The continuous variables will be presented as their means and standard deviations according to statistical distribution (Shapiro–Wilk test), or as medians and interquartile ranges. The categorical parameters will be expressed as the number of subjects and associated percentages. The patients will be described and compared at inclusion according to the following variables: compliance with the eligibility criteria, epidemiological characteristics, clinical characteristics, biological characteristics and nutrition characteristics. Comparison between the two randomised groups concerning the primary endpoint will be analysed using the chi-squared test or exact Fisher's exact test, when appropriate. A logistic regression model will be performed to consider adjustments on baseline factors such as centre stratification parameters. Comparisons concerning other categorical criteria (*eg*. occurrence of GVHD or mucositis) will be realised as indicated previously. Comparisons between the two arms regarding quantitative criteria (*eg*. duration of nutrition intervention) will be assessed using Student's *t*-test (or the Kruskal–Wallis test, when appropriate). Initial number of days of hospitalization, number of transfers to the ICU and number of days of rehospitalization will be compared between groups, according to their statistical distribution using Poisson or negative binomial regressions. The censored data as survival rates (overall and progression-free, at one year) will be estimated by the Kaplan–Meier method and compared between randomisation groups using the log-rank test for univariate analysis and Cox's proportional-hazards regression for multivariate analyses. Finally, the follow-up of the clinical and biological parameters (nutritional: *i.e.* weight, body-mass index, brachial circumference, albumin and transthyretin; and inflammatory: C-reactive protein) collected at various times, will be analysed using mixed models 1) in order to take into account within and between patient variability (random-effects: slope and intercept) and 2) in order to study fixed effects : group, time and their interaction.

## Discussion

The aim of this study is to evaluate the impact of nutritional support modalities during allo-HSCT on mortality and early immunological and infectious toxicities. Indeed, although the consequences of malnutrition are well known and nutritional support is recommended in the allo-HSCT setting, there is a lack of data on the best method of artificial nutrition, i.e. enteral or parenteral (ASPEN, European society of clinical nutrition and metabolism (ESPEN), SFNEP). The few data in the literature assume that EN may decrease early complications in allo-HSCT, notably infections and immunological issues, and decrease D100 mortality [23,24].

Rare data available in retrospective studies in this "enteral nutrition / allo-HSCT" field mentioned event rates for overall survival at D100 with myeloablative conditioning regimens respectively at 33% in PN group vs 8% in EN group, suggesting that 75% of early deaths could be avoided with EN. Even if these results can appear counter-intuitive considering solely correctable nutritional problems, it could be partially explained by recent scientific data suggesting that route of nutrition administration could impact on more than nutritional status. As instance, and even if these issues are not directly explored by our study, there are strong data suggesting an impact of nature and route of nutritional administration on dysbiosis [28-32], which has recently been shown to directly impact on post allo-HSCT survival [33].

The main evaluation criterion in this study is D100 mortality, which is a strong marker of early allo-HSCT toxicity, classically used in allo-HSCT trials; secondary criteria will evaluate haematological and nutritional outcomes. Therefore, we may be in the best position to evaluate the impact of EN on early allo-HSCT toxicity, and haematological and nutritional outcomes. Moreover, we have anticipated all the expected pitfalls related to allo-HSCT's intrinsic complexity and heterogeneity. In spite of these precautions, few limitations still remain: these are listed below.

EN in allo-HSCT needs the use of specific and adapted protocols, which have shown their efficacy and security in some centres [23-25]. The daily practical application of these precise and validated procedures requires experience. There is a risk that a lack of experience in some centres regarding NE administration after allo-HSCT may result in non-optimal use of EN, and lead to under-estimation of EN efficacy and tolerance. To avoid this risk, we have trained medical and paramedical teams in the main EN procedures, and have encouraged them to use EN before this trial began.

Furthermore, with the development and rapid spread of reduced-toxicity myeloablative conditioning regimens, early toxicity after allo-HSCT has decreased since this trial was first designed, reducing the D100 mortality to ~10% in the population with haematological malignancy [34]. Thus, toxicity in the PN group could be lower than expected according to our initial hypothesis: this may lead to an insufficient statistical power to demonstrate EN superiority.

## Trial status

The local ethical committee (CPP Sud Est VI) agreement was obtained in December 2012. The French national authorities (ANSM) agreement was obtained in July 2013. NEPHA recruitment has been ongoing since November 2013. Study completion date is estimated at June 2017. Clinical trials gov number: NCT01955772.

## Abbreviations

Allo-HSCT: Allogeneic hematopoietic stem cell transplantation
ANSM: French National Health Authorities
ASPEN: American society of enteral and parenteral nutrition
EN: Enteral nutrition
ESPEN: European society of clinical nutrition and metabolism
GVHD: Graft-versus-host disease
D100 mortality: Mortality at day 100
ICU: Intensive care unit
NEPHA: Comparison of Nutritional support with Enteral or Parenteral procedures in patients with Hematological malignancies undergoing Allogeneic stem cell transplantation
PN: Parenteral nutrition
SFNEP: French-speaking society of clinical nutrition and metabolism
